# Enhancing clinical reasoning skills through tailored CPC in pathology laboratory instruction

**DOI:** 10.3389/fmed.2025.1566097

**Published:** 2025-07-25

**Authors:** Shanshan Li, Xufei Tan, Jie Fang, Jingyin Dong

**Affiliations:** School of Medicine, Hangzhou City University, Hangzhou, China

**Keywords:** clinical reasoning skills, pathology laboratory instruction, foundational medical courses, course-specific CPC teaching, early-stage students

## Abstract

**Background:**

In medical education, fostering students’ clinical reasoning skills is crucial for assessing their clinical proficiency. However, developing a Clinical Pathological Conference (CPC) model tailored to the unique needs of early-stage learners remains an intriguing avenue for exploration.

**Methods:**

This study aimed to enhance the clinical reasoning and critical thinking abilities of early-stage medical students in pathology laboratory instruction through the introduction of a customized CPC teaching method. A total of 279 undergraduate students from the clinical medicine program at Hangzhou City University participated in the study. The 2021 cohort (*n* = 139) received traditional teaching methods, while the 2022 cohort (*n* = 140) was taught using a reformed CPC case-based approach. Evaluations included post-class case analysis scores, final examination scores, overall evaluation scores, and an online survey to assess feedback on teaching content, student engagement, and learning outcomes.

**Results:**

Students who underwent the CPC case teaching method exhibited higher levels of enthusiasm, participation, learning efficiency, and clinical reasoning abilities compared to those following traditional teaching methods. Quantitative results also showed improvements in post-class case analysis and final examination scores. Qualitative feedback indicated that the method was generally well-received, although some students suggested improvements in group collaboration and personalized guidance.

**Conclusion:**

The course-specific CPC teaching method effectively enhances students’ learning enthusiasm, classroom participation, learning efficiency, and clinical thinking abilities in pathology laboratory instruction. These findings pave the way for future research to explore the design and implementation of CPC methods in other foundational medical courses and to evaluate their effectiveness.

## 1 Introduction

Within the realm of medical education, the cultivation of clinical reasoning skills is regarded as a cornerstone of clinical practice, serving as a pivotal benchmark for assessing a physician’s clinical acumen (1, 2). A key pedagogical challenge lies in how to effectively enhance these critical thinking abilities among students. One widely acknowledged approach is the integration of the Clinical Pathological Conference (CPC) within the curriculum (3). Originated at Harvard Medical School (4), the CPC facilitates collaborative discussions between pathologists and clinicians regarding the diagnostic and therapeutic processes of deceased patients. These sessions aim to ascertain the concordance between clinical and pathological diagnoses, evaluate the appropriateness of the treatment regimen, and scrutinize the implications of any medical errors, including potential accountability issues (5, 6). Additionally, they assist in elucidating the underlying causes and mechanisms of patient mortality. However, the CPC is typically most beneficial for advanced students who have already completed foundational clinical coursework. Hence, developing a CPC model that resonates with the unique needs of early-stage learners presents an intriguing avenue for further exploration.

As a fundamental pillar of medical education, pathology delves into the study of disease etiology, mechanisms, pathological transformations, outcomes, and prognoses, acting as a vital bridge between basic science and clinical practice (7, 8). Laboratory instruction in pathology is instrumental in enabling students to gain a direct understanding of diseased organs and tissues, while fostering a deeper integration of theory with practical application through specimen examination and case-based discussions. However, conventional pathology laboratory teachings tend to emphasize observational learning over the development of clinical reasoning skills, thereby limiting students’ active engagement and critical thinking abilities, which are essential for effective learning and professional growth (9).

Given these considerations, the current pedagogical reform aims to introduce a tailored set of intermediate-level cases specifically designed for early-stage students. The objective is to encourage a comprehensive application of interdisciplinary foundational knowledge and core pathological insights, facilitating a thorough analysis of cases from a pathological perspective, promoting critical thinking, and enabling the formulation of diagnostic conclusions. This study investigates the implementation of adapted CPC case teaching within pathology laboratory instruction, evaluating its efficacy and inherent advantages. To achieve this, the study aims to provide substantial evidence and innovative ideas to support the ongoing transformation of teaching methodologies in pathology and other bridging medical courses.

## 2 Materials and methods

This was a prospective study involving 279 undergraduate students enrolled in the clinical medicine program at Hangzhou City University (Hangzhou, China). The inclusion criteria comprised students who had completed the same foundational courses prior to enrollment in the current course, with comparable admission scores and academic performance in prerequisite subjects—particularly, Human Anatomy and Histology–Embryology (see Table 1). Students who took a leave of absence or failed to complete all instructional activities were excluded from the final analysis. Participants were assigned to one of the two groups: the 2021 cohort received traditional teaching methods (*n* = 139), while the 2022 cohort was taught using an innovative, case-based integrated teaching approach (*n* = 140). Prior to the commencement of the study, informed consent was obtained through active and voluntary engagement. All students were provided with detailed information regarding the general purpose of the study, data usage, anonymity, and their right to withdraw at any time. Written informed consent was then obtained from each participant before enrollment. Importantly, to minimize potential bias associated with group awareness, students were not explicitly informed whether they belonged to the experimental or control group, nor were they made aware of the specific differences in instructional design between the two cohorts.

**Table 1:** Participant characteristics.

Number of participants (percentage)		
Sex	2021 (*n* = 139)	2022 (*n* = 140)
Male	77 (53.79%)	74 (52.50%)
Female	62 (45.83%)	66 (46.81%)
Admission scores/750	596.2 ± 4.83	597.6 ± 3.84
Histology and embryology scores/100	69.88 ± 14.50	73.46 ± 12.07
Human anatomy scores/100	68.07 ± 13.28	69.66 ± 12.17

The study was approved by the Ethics Committee and Institutional Review Board (IRB) of the School of Medicine at Hangzhou City University.

### 2.1 Design and usage principles of the CPC

To ensure the scientific rigor and effectiveness of CPC cases, the teaching team adopted a systematic approach consisting of four interconnected steps: goal-oriented design, graded validation, dynamic adjustment, and teacher review.

#### 2.1.1 Case design process

##### 2.1.1.1 Objective and difficulty alignment

Each case is meticulously aligned with both knowledge objectives (e.g., pathologic mechanisms and diagnostic criteria) and competency objectives (e.g., clinical reasoning and information integration) from the curriculum. This alignment forms a three-tier difficulty system (see Supplementary File for examples):

Basic level (CPC1): Knowledge objectives account for 90% of the focus (e.g., identifying typical pathological features) and application objectives account for 10% (e.g., proposing differential diagnoses based on pathological changes and clinical presentations), both of which are suitable for the initial stages of teaching.
Intermediate level (CPC2-6): Knowledge objectives account for 80% and application objectives account for 20%, both of which are designed to reinforce skills and develop the ability to link theory with clinical practice.
Advanced level: Knowledge objectives account for 70% and application objectives account for 30%, both of which are aimed at further enhancing capabilities and reserving high-level comprehensive assessment scenarios (not used in this round).

##### 2.1.1.2 Pre-testing and dynamic calibration

The CPC cases were pre-tested by senior clinical medicine students to gather feedback on aspects such as the amount of information, logical complexity, and diagnostic challenges. Based on this feedback, the cases were revised and optimized to better meet educational goals.

##### 2.1.1.3 Collaborative teacher review

Three external pathology teachers were invited to independently assess the difficulty level of the cases using a double-blind scoring method. A consistency rate of over 90% was required to ensure the accuracy and fairness of the case difficulty assessments.

#### 2.1.2 Implementation process

In this round of teaching, CPC1 was classified as basic-level difficulty, while CPC2-CPC6 were categorized as intermediate-level difficulty. The teaching process followed the cognitive principle of “from simple to complex,” gradually introducing cases of increasing difficulty. This approach aims to help students progressively master knowledge and enhance their application skills.

### 2.2 Design of the teaching process

In the teaching design, the control group (2021 grade) had no clinical case discussions during class but received two case analyses (Cases 1 and 2) after class. The experimental group, in addition to these, conducted four additional case analyses (Cases 3 to 6). All other teaching activities remained the same.

**Figure 1:**
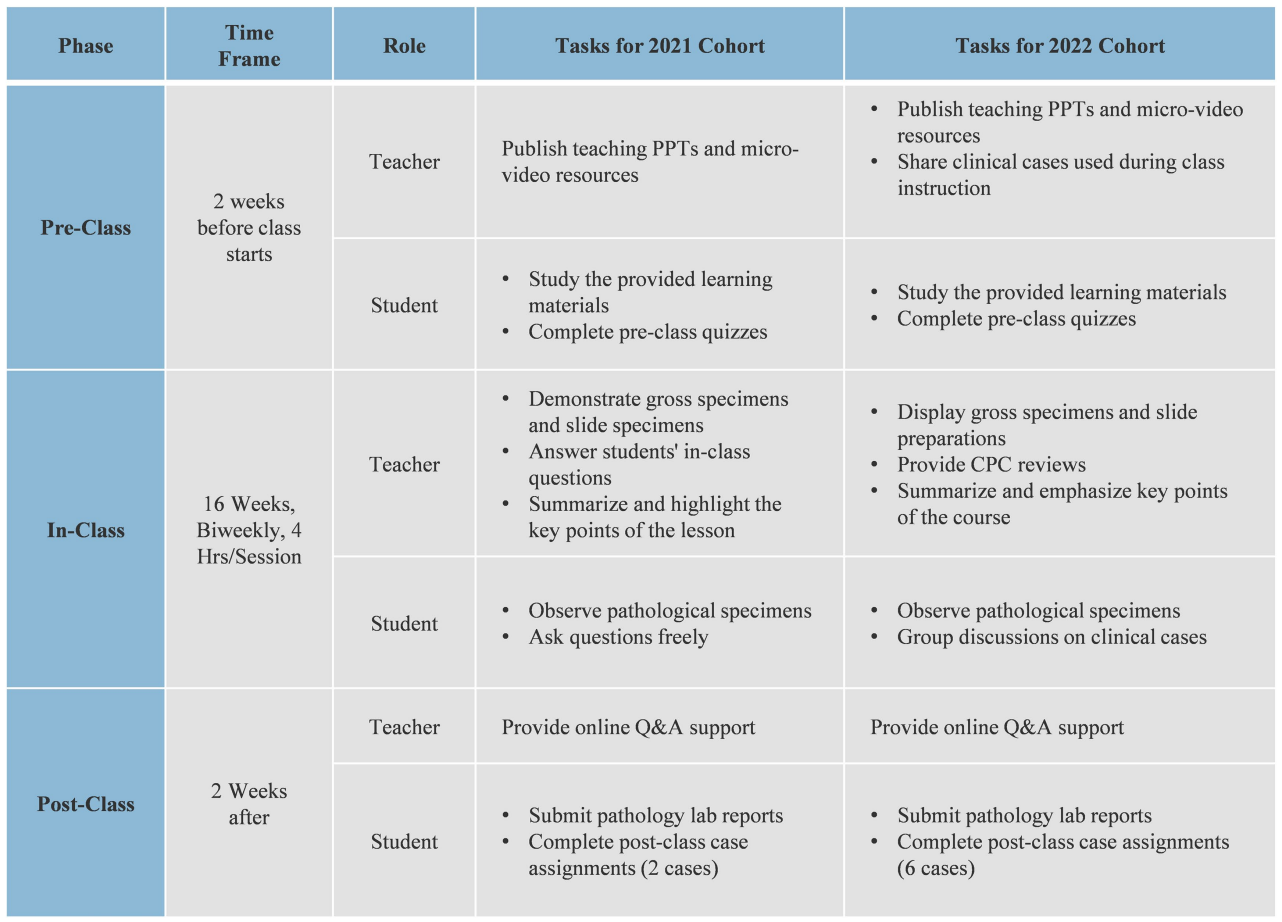
Pathology laboratory teaching implementation plan.

The CPC-integrated teaching design is as follows: Teaching activities are divided into pre-class, in-class, and post-class phases. In the pre-class phase, teachers release preparatory tasks, including annotated PPTs, micro-video lectures, and clinical cases for discussion, 2 weeks in advance; students complete their pre-class learning based on these tasks. During the in-class phase, the activities include four parts: instructor-led explanations, observation of pathological specimens and slides, clinical case discussions, and instructor summaries, along with homework assignments. First, the instructor checks students’ understanding of the preparatory material through in-class quizzes or tests. Then, the instructor delivers the lesson based on the curriculum and students’ responses, focusing on common queries and specific pathological specimens. Next, students observe the specimens and write reports based on the provided materials. In the CPC discussion session, students discuss assigned topics in groups, consolidating their insights. One representative from each group presents their findings, covering diagnoses and supporting evidence, as well as the progression of the condition. The instructor provides feedback, emphasizing correct diagnostic procedures and addressing unresolved questions. Finally, the instructor summarizes the key points and assigns homework to reinforce the learned concepts. After class, the instructor assigns clinical case assignments and provides answers to questions; students submit their laboratory reports and case assignments on time and complete peer evaluations of the assignments (see Figure 1).

### 2.3 Data evaluation

The evaluation of teaching outcomes includes three components: post-class case analysis scores, final examination scores, and overall evaluation scores. Specifically, the overall evaluation (100%) is calculated as follows: online course learning and testing (20%) + class performance (20%) + laboratory report scores (10%) + case analysis assignment scores (20%) + final examination (30%). Among these, the case analysis assignments are particularly significant, as they not only assess students’ understanding of pathology and its clinical correlations but also emphasize the application of critical thinking in clinical reasoning. Students are expected to integrate theoretical knowledge with clinical information, formulate and evaluate diagnostic hypotheses, and justify their conclusions based on evidence—all of which represent core aspects of critical thinking in medical education. The final examination covers the application of basic pathological knowledge and case analysis skills, with a particular focus on students’ clinical reasoning and decision-making abilities under time constraints and pressure, closely simulating real-world clinical scenarios. The overall evaluation score comprehensively reflects students’ academic performance and competence development throughout the entire learning process. Additionally, a survey was conducted through the “Study at City College” platform to collect student feedback. The survey included 10 questions assessing the evaluation of teaching content, interest and engagement in learning, learning outcomes and gains, using a 4-point scale for satisfaction assessment. The survey also gathered students’ opinions on the format and scheduling of clinical case discussions in class, the difficulty of the cases, and suggestions for improvement, providing guidance for refining future teaching methods.

### 2.4 Statistical analysis

This study includes both descriptive and inferential statistical analyses. Data were processed using GraphPad Prism 7.0 software and are presented as mean ± standard deviation (SD). To assess the distribution characteristics of the data, normality tests were considered in conjunction with Q-Q plots to make a comprehensive judgment. This was because normality tests can be overly sensitive under conditions of large sample sizes. The results indicated that the data approximately followed a normal distribution. Based on this finding, inter-group comparisons were conducted using a two-tailed independent samples *t*-test. In addition to reporting statistical significance (*p* < 0.05), this study also calculated Cohen’s d effect size to further evaluate the practical significance of the observed differences. According to Cohen’s criteria, the following standards were used to interpret the magnitude of the effect: |*d*| = 0.2 indicates a small effect, |*d*| = 0.5 indicates a medium effect, and |*d*| = 0.8 indicates a large effect.

## 3 Results

### 3.1 Post-class clinical case test scores

To evaluate the effectiveness of this pedagogical reform, the control group from the class of 2021 completed analysis tasks for two cases (Cases 1 and 2) in their post-class case study assignments, whereas the experimental group from the class of 2022, in addition to these, also completed analyses for four new cases (Cases 3 to 6), aimed at comprehensively assessing students’ understanding of pathological diagnosis and the progression of diseases in clinical scenarios.

**Figure 2:**
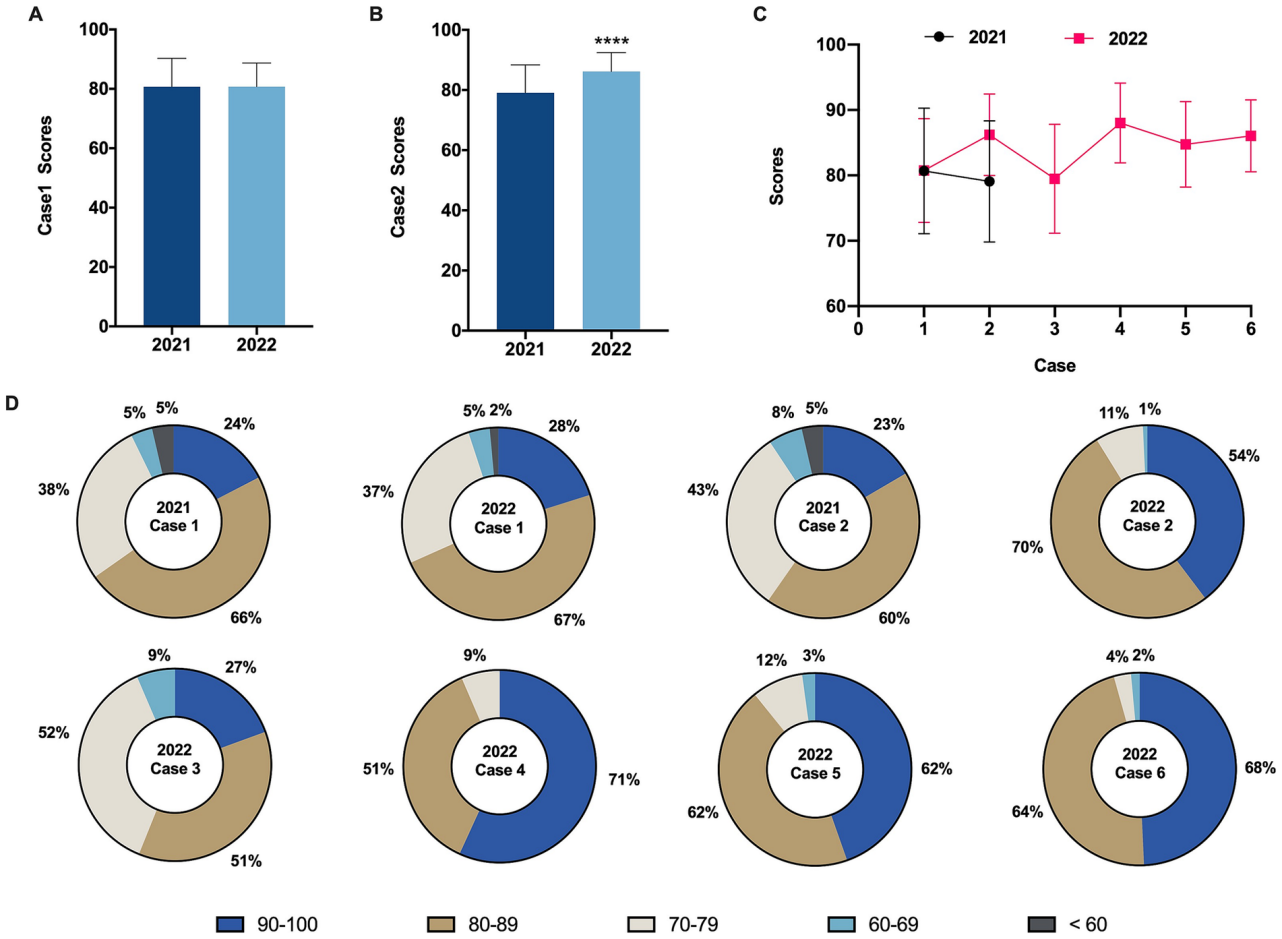
Comparison of post-intervention clinical case test scores. **(A)** Comparison of Case 1 scores before and after intervention. **(B)** Comparison of Case 2 scores before and after intervention. **(C)** Performance of the control group (2021 grade) and the experimental group (2022 grade) across different cases. **(D)** Proportion of students within five grading categories (100–90, 89–80, 79–70, 69–60, less than 60) for case scores in the control group (2021 grade) and the experimental group (2022 grade). The omission of certain score brackets in the graph represents zero occurrences within those ranges. Data are presented as mean ± SD. ^∗∗∗^*p* < 0.001.

From the scores in Case 1, there was no significant difference between the performance of the control group (class of 2021) with a score of 80.70 ± 9.62 and the experimental group (class of 2022) with a score of 80.76 ± 7.94 (Figure 2A, |*d*| = 0.007). However, in Case 2, the experimental group’s score (86.21 ± 6.22) was significantly higher than that of the control group (79.09 ± 9.28) (Figure 2B, ^∗∗∗^*p* < 0.001, |*d*| = 0.899). Moreover, it is worth noting that the experimental group also achieved relatively higher scores in the subsequent four cases (Cases 3 to 6), with average scores of 79.48 ± 8.33, 88.02 ± 6.10, 84.75 ± 6.52, and 86.05 ± 5.49, respectively (Figure 2C). These results suggest that, without extensive training in clinical case analysis, there was no significant difference in performance between the two groups; however, as the amount of training increased, the experimental group demonstrated a notably enhanced ability in diagnosing and analyzing cases.

In addition to comparing the average scores across the cases, we conducted a statistical analysis of the number of students falling into five different score brackets (90–100, 80–89, 70–79, 60–69, < 60) for each case (Figure 2D). The results showed that the distribution of scores across the five brackets was similar for both groups in Case 1. However, in Cases 2, 4, and 6, the proportion of students in the experimental group (class of 2022) achieving scores in the 90–100 and 80–89 brackets increased significantly. This finding indicates that continuous practice through classroom case studies can enhance the accuracy and excellence rate in pathological diagnostic analysis, thereby deepening students’ understanding and application of theoretical knowledge.

### 3.2 Final examination scores and overall evaluation scores

**Figure 3:**
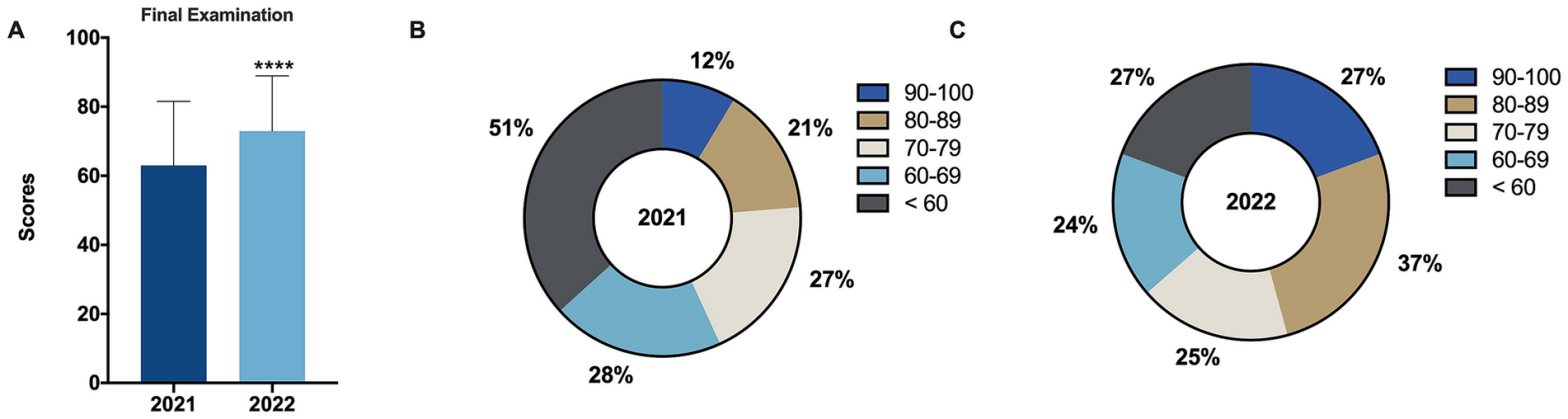
Comparison of final examination scores before and after intervention. **(A)** Comparison of final examination scores for the control group (2021 grade) and the experimental group (2022 grade) before and after intervention. **(B,C)** Proportion of students within five grading categories (100–90, 89–80, 79–70, 69–60, and < 60) for final examination scores for the control group (2021 grade) **(B)** and the experimental group (2022 grade) **(C)**. Data are presented as mean ± SD. ****p < 0.0001.

As shown in Figure 3A, the average final examination scores for the pathology practical course were 62.99 ± 18.53 and 72.94 ± 15.97 for the 2021 grade and the 2022 grade, respectively, with a significant difference between the two (Figure 3A, ****p < 0.0001, |*d*| = 0.575). Specifically (Figures 3B,C), the proportions of students scoring in the 90–100 range were 8.63% (12/139) and 19.29% (27/140) for the 2021 grade and the 2022 grade, respectively; while in the 80–89 range, the proportions were 15.11% (21/139) and 26.43% (37/140) for the 2021 grade and the 2022 grade, respectively. These findings indicate that the proportion of students in the 2022 grade scoring in the 80–89 and 90–100 ranges was significantly higher than that in the 2021 grade, suggesting that the use of CPC-style case teaching methods helps in cultivating students’ clinical thinking abilities by linking theory with practice.

**Figure 4:**
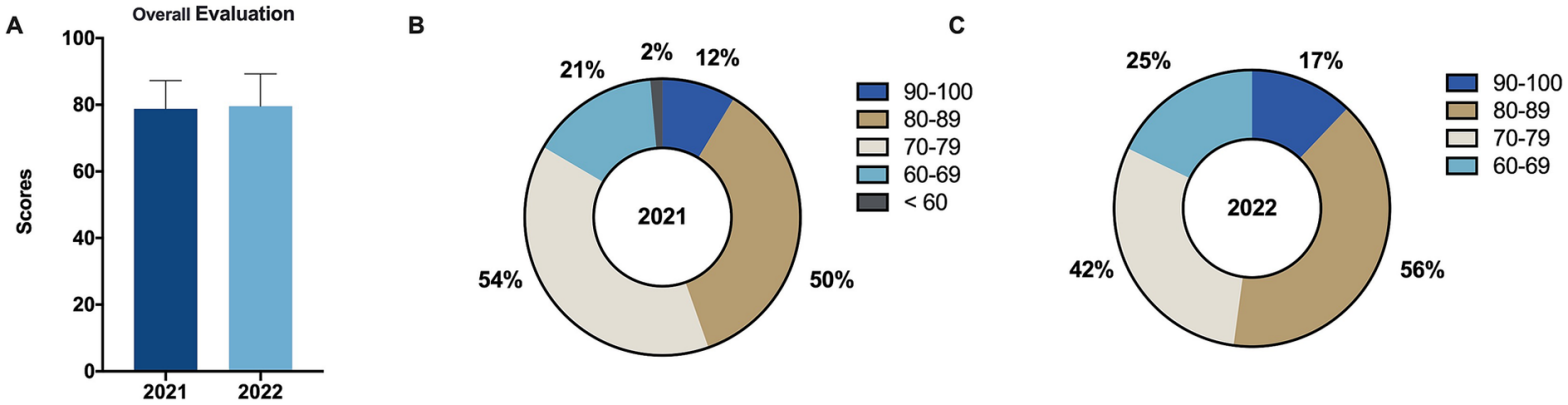
Comparison of overall evaluation scores before and after intervention. **(A)** Comparison of overall evaluation scores for the control group (2021 grade) and the experimental group (2022 grade) before and after intervention. **(B,C)** Proportion of students within five grading categories (100–90, 89–80, 79–70, 69–60, and < 60) for overall evaluation scores for the control group (2021 grade) **(B)** and the experimental group (2022 grade) **(C)**. Data are presented as mean ± SD.

Similar to the aforementioned results, the average overall evaluation score for the 2022 grade was 79.56 ± 9.72, which was slightly higher than the 2021 grade’s score of 78.81 ± 8.44, but this difference was not significant and had no statistical meaning (Figure 4A, |*d*| = 0.082). However, when comparing the proportions of students in each score bracket between the two groups (Figures 4B,C), it can be observed that the 2022 grade had slightly higher proportions in the 90–100, 80–89, and 60–69 score brackets at 12.06% (17/140), 39.72% (56/140), and 17.73% (25/140), respectively, compared to the 2021 grade’s score brackets at 8.63% (12/139), 35.97% (50/139), and 15.11% (21/139), respectively.

### 3.3 Satisfaction questionnaire

To evaluate the effectiveness of this teaching approach, a survey was conducted at the end of the second semester of the 2023–2024 academic year, targeting 140 students from the 2022 grade, with 132 valid responses collected. As shown in Table 2, 97% of the respondents believed that integrating clinical case discussions into the pathology laboratory course was necessary. Additionally, 95% found the clinical cases discussed during the semester to be highly practical and relevant, with no students expressing dissatisfaction with this teaching content. Students demonstrated high levels of engagement in the CPC case teaching. Among the respondents, 89% expressed interest in the CPC case teaching, and 83% believed they actively participated in CPC discussions. Furthermore, the survey results indicated that over 90% of the students felt that clinical case discussions significantly enhanced their grasp of theoretical knowledge, clinical knowledge, the ability to integrate theory with practice, and clinical thinking skills. Additionally, 89% reported a notable improvement in their comprehensive analytical skills, and 80% noted a marked enhancement in their teamwork abilities.

**Table 2:** Satisfaction survey based on tailored CPC teaching.

Survey content	Question	Answers (Percent)			
		Agree	Somewhat agree	Neutral	Disagree
Evaluation of Teaching Content	1. How necessary do you think it is to incorporate clinical case discussions?	74.58%	21.55%	3.87%	0.00%
	2. How would you evaluate the practicality and relevance of the content discussed in the case studies?	69.35%	26.80%	3.85%	0.00%
Interest and Participation	3. How interested are you in the case discussions?	49.50%	38.88%	11.62%	0.00%
	4. How engaged were you in the case discussions?	49.45%	33.63%	16.92%	0.00%
Learning Outcomes and Gains	5. Do you feel that your grasp of theoretical knowledge has improved through case discussions?	53.98%	40.64%	5.38%	0.00%
	6. Do you feel that your understanding of clinical knowledge has improved through case discussions?	50.95%	39.83%	9.22%	0.00%
	7. How effective do you think case discussions have been in promoting the integration of theoretical knowledge with practical application?	59.35%	32.95%	7.70%	0.00%
	8. How effective do you think the integration of clinical case discussions has been in improving your clinical thinking skills?	61.64%	29.88%	8.48%	0.00%
	9. Do you feel that your comprehensive analytical skills have improved through case discussions?	47.87%	41.40%	10.73%	0.00%
	10. Do you feel that your teamwork abilities have improved through case discussions?	48.65%	30.60%	17.65%	3.10%

## 4 Discussion

Clinical thinking is the foundation of disease diagnosis and treatment and is critical in determining a physician’s medical proficiency (10–12). The ability to engage in clinical thinking involves a rational process of revealing the essence of a disease through its phenomena (13, 14). The CPC is a comprehensive introduction to clinical cases that includes patient history, laboratory tests, biopsy results, treatment, and autopsy findings to determine pathological diagnoses and causes of death (15, 16). This approach is fundamental for clinical decision-making, including therapy. The CPC is recognized as an effective method to enhance students’ clinical thinking abilities (16), applicable not only for training medical students and young physicians but also for improving the diagnostic and therapeutic skills of experienced senior doctors, thereby helping to avoid misdiagnoses (17, 18).

However, for second-year medical students who have completed foundational courses such as anatomy, histology, embryology, physiology, and biochemistry, but have yet to be exposed to clinical courses, the standard CPC teaching presents certain challenges. Therefore, to adapt to the knowledge level and learning needs of lower-year medical students, the teaching team has adapted clinical cases and developed a unique CPC-style case discussion course. This course aims to explore the effectiveness of this teaching method in fostering students’ critical thinking and clinical thinking skills, as well as their mastery of pathology knowledge and overall satisfaction with the course.

First, the changes in performance scores from clinical case analysis indicate that students from the 2022 grade showed significant improvement after receiving a CPC case-based instruction. In particular, in handling Case 2 and subsequent cases, the 2022 grade performed notably better than the 2021 cohort. This finding suggests that, as CPC case teaching progressed, students’ abilities in case analysis and diagnosis were significantly enhanced. Much of this improvement can be attributed to the methods used during CPC discussions, where students identify pathological changes, rule out misleading diagnostic information, and establish connections between suspected diagnoses, etiologies, and clinical presentations to verify correct diagnoses. This method effectively cultivates students’ critical thinking and logical analysis skills, thereby enhancing the accuracy of pathological diagnoses. These results directly confirm the efficacy of the CPC case teaching in promoting the development of students’ clinical thinking abilities. This finding is also consistent with the results of Engelberg (17) and Bender et al. (18), both of whom reported that CPC-style case discussions significantly enhance students’ understanding of pathophysiological mechanisms. Furthermore, it supports Yu’s assertion that an innovative CPC instructional design integrating traditional lectures, online learning, and a flipped classroom model facilitates the effective integration of basic and clinical knowledge in oral diagnostics (3).

Second, the average scores of the 2022 grade in the final written examination were significantly higher than those of the 2021 grade, with a noticeable increase in the proportion of high-scoring students. Academic performance is a key indicator of students’ mastery of knowledge and their ability to apply it. The final examination in pathology covers general and specific topics, including case-type questions and the identification of pathological slides and gross specimens, aiming to comprehensively assess students’ foundational knowledge, clinical thinking skills, and the ability to integrate knowledge. Thus, it further supports the effectiveness of the CPC case teaching approach tailored to the course. However, there was no significant difference in the overall pathology laboratory performance scores between students in the 2022 grade and those in the 2021 grade. This outcome may be attributed to the fact that the overall score for the 2022 grade included evaluations from four CPC case teaching sessions, which were assessed using stricter criteria. In contrast, the 2021 grade’s classroom questioning component was easier for to score highly in, resulting in relatively lower classroom performance scores for the 2022 cohort. Ultimately, this led to no significant difference in the final total scores.

The survey results regarding students’ perceptions of the CPC case-based teaching approach were consistent with previous studies, showing that the majority of students positively affirmed the necessity of CPC instruction, as well as the practicality and relevance of the clinical cases used. They also reported high levels of engagement and perceived learning gains (3, 19). These findings confirm that CPC case teaching achieves its goal of progressively enhancing training through interactive methods, while emphasizing the importance of cultivating students’ clinical thinking abilities and critical spirit (20). This demonstrates the significant value of applying this method more widely within foundational medical education disciplines.

## 5 Limitations

Based on feedback from teacher and student surveys conducted during the implementation of teaching reforms, a series of further improvements is needed in future teaching practices. For example, survey results indicate that 18% of students felt that CPC case teaching did not significantly enhance their teamwork abilities, while another 3% found this method to be almost ineffective. This suggests that, in subsequent teaching designs, there should be more detailed guidance on group formation principles and collaborative learning mechanisms within groups to strengthen students’ teamwork skills. Notably, 8% of students rated the CPC case teaching as mediocre, a proportion similar to those who performed poorly on the final assessments. These students might face issues such as insufficient motivation, poor study habits, or inadequate preparation before class, leading to difficulties in understanding and applying the CPC case teaching. This finding indicates a need to provide these students with more personalized guidance and support to improve their mastery of theoretical knowledge, thereby enhancing their learning experience and outcomes.

Despite our efforts to control for potential confounding variables, several limitations should be acknowledged. First, although students were not explicitly informed of their group assignment to minimize awareness-related behavioral changes (i.e., the Hawthorne effect), the fact that they signed informed consent forms and knew they were participating in an educational research project may have still influenced their behavior—though not necessarily in a systematic or predictable way. Second, although the same teaching team and standardized teaching materials were used throughout the study, minor variations in instructors’ enthusiasm across different semesters may have influenced students’ levels of engagement and learning outcomes. Finally, while a “neutral” option was included in the satisfaction survey to reduce response bias, the results showed an overwhelming tendency toward positive responses (e.g., 0% selected “disagree”), which may indicate social desirability bias or suggest that some items were phrased in a leading manner. Future studies should aim to improve questionnaire design by using more neutrally worded items and incorporating complementary assessment methods to enhance the validity and objectivity of student feedback.

## 6 Conclusion

The results of this study indicate that the course-specific CPC teaching method can effectively enhance students’ learning enthusiasm, classroom participation, learning efficiency, and clinical thinking abilities in pathology laboratory instruction. However, during implementation, there remains room for improvement in areas such as group learning, collaboration, and individualized guidance. Future research could further explore the design and implementation of course-specific CPC methods, as well as evaluate their effectiveness in other foundational medical courses.

## Data Availability

The original contributions presented in the study are included in the article/Supplementary material, further inquiries can be directed to the corresponding author.
