# Supplementary material for: Enhancing clinical reasoning skills through tailored CPC in pathology laboratory instruction
Source: Front Med (Lausanne). 2025 Jul 25;12:1566097. doi: 10.3389/fmed.2025.1566097 (PMC12331614; doi:10.3389/fmed.2025.1566097)
Supplement: Supplementary file 1 [file Data_Sheet_1.pdf]

**CPC Example + Teaching Process**

**[Clinical History Summary]**

Male, 72 years old. Due to rheumatic heart disease with mitral stenosis and regurgitation, the patient has experienced palpitations, shortness of breath, and bedridden status for more than half a year, accompanied by coughing up pink frothy sputum. Auscultation reveals widespread rales in both lungs, with obvious diastolic and systolic murmurs in the apex area. Jugular veins are distended, liver is palpable four fingers below the costal margin with mild tenderness, and there is significant edema in both lower extremities and back. One month ago, while attempting to defecate in a squatting position due to difficulty in passing stool, the patient suddenly developed respiratory distress, pallor, groaning loudly, profuse sweating, rapidly became unconscious and flaccid, and spontaneous breathing ceased within minutes. After timely resuscitation using a mechanical ventilator and thoracotomy, the patient gradually recovered. However, subsequently, the patient experienced right chest pain that worsened during respiration, with friction rub audible locally and hemoptysis. Ten days ago, due to severe pulmonary infection, treatment was ineffective, and the patient ultimately died from cessation of respiration and heartbeat.

**[Autopsy Summary]**

**Heart**: Confirmed as chronic rheumatic heart disease with mitral stenosis and regurgitation. The apex is blunt and rounded, with marked enlargement and hypertrophy of the left and right ventricles. A moist, glossy, dark red clot-like solid can be completely removed from the cardiac chambers, matching their shape.

**Lungs**: Both lungs are swollen and enlarged, appearing dark red. On sectioning, there is a large amount of bloody frothy fluid. In the lateral region of the right middle lobe, there is a wedge-shaped dark red consolidation lesion, relatively firm, with elevated local surface and rough pleura, appearing grayish-yellow. There are scattered foci of lobar pneumonia in both lungs, which have coalesced

into patches in the lower lobes. Small branches of the pulmonary artery near the consolidation lesion contain cylindrical dark red clot-like solids. Microscopically, diffuse dilation and engorgement of alveolar capillaries and small veins are observed, along with fibrous tissue proliferation. Alveolar spaces contain pale red homogeneous fluid interspersed with bubbles, red blood cells, and macrophages containing brownish-yellow granules. Neutrophilic exudates are visible in some bronchioles and surrounding alveolar spaces. The consolidation lesion in the lateral region of the right middle lobe shows disappearance of various cell nuclei in alveoli but retains the outline of alveolar structures, filled with numerous red blood cells and their breakdown products. The lung tissue near the consolidation lesion shows congestion and leukocyte infiltration.

**Right Internal and External Iliac Veins and Common Iliac Vein**: The lumens contain dark red cylindrical clot-like solids. The ends are moist and glossy, free from the local intima; the central portion is drier, with a reddish-white appearance, and adheres tightly to the local intima.

**Liver**:

Gross specimen image

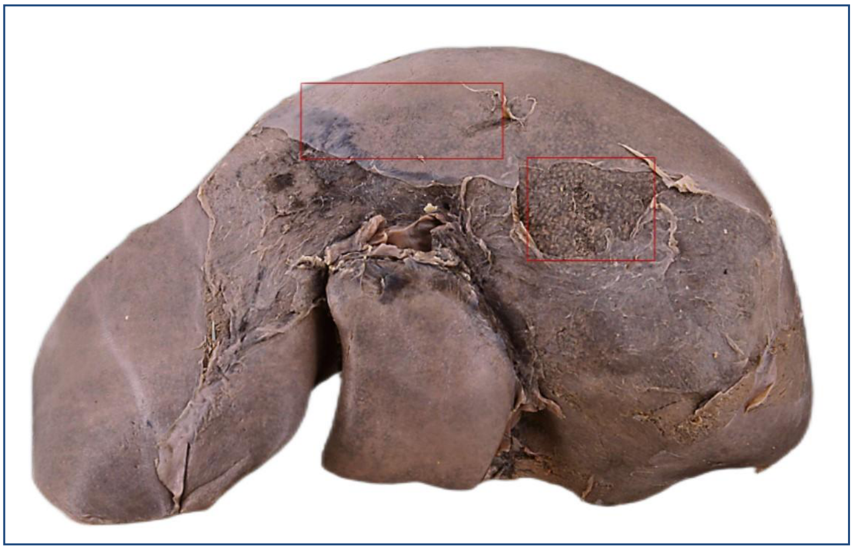

microscopic features images

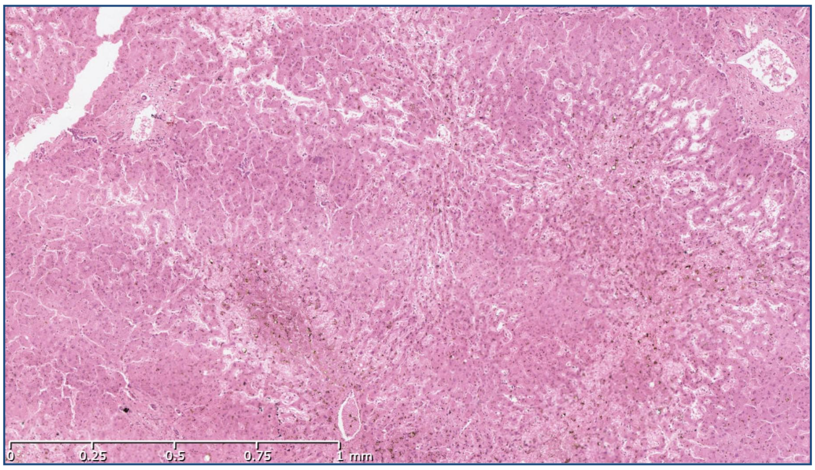


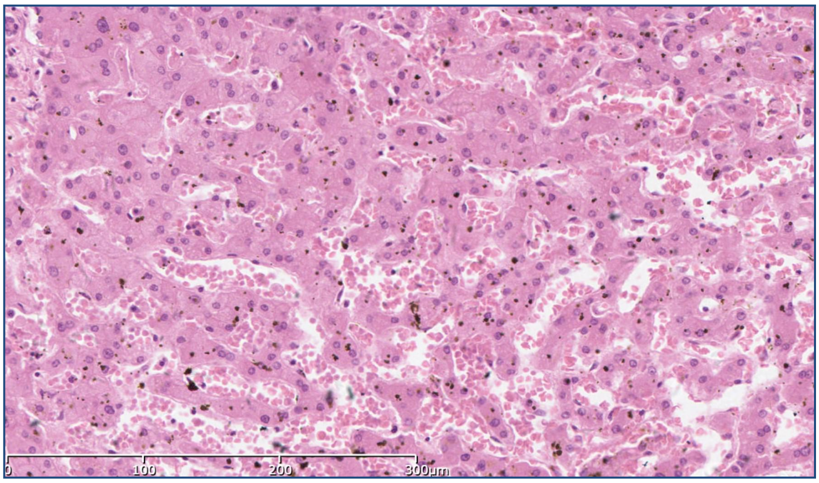


**[Discussion Questions]**

1. What is the nature of the dark red solid materials found in the cardiac chambers, pulmonary artery branches, and iliac veins at autopsy? How can they be differentiated?
2. What caused the formation of the dark red solid material in the patient's iliac vein? What impact does this lesion have on the body?
3. Based on this case, summarize the similarities and differences between thrombosis and embolism leading to arterial ischemia.
4. Describe the pathological diagnosis of the patient's liver and its gross and microscopic features. How can you determine whether the round vacuoles in hepatocytes are water, lipid droplets, or glycogen vacuoles?

5. What lesions are present in the patient's heart? What are the pathological manifestations of chronic rheumatic heart disease? What are the characteristic pathological changes of rheumatism, and were these changes observed in this patient's autopsy?
6. What lesions are present in the patient's lungs? What are the diagnostic criteria? Explain the causes of various lesions. Is the pulmonary congestion acute or chronic? How can it be distinguished?
7. Based on the main findings of this autopsy, attempt to make a systematic pathological diagnosis and explain the primary diseases and cause of death.
8. Using the autopsy findings, explain the relevant clinical manifestations before the patient's death.
9. Create a mind map illustrating the progression and development of the patient's condition, highlighting the relationships between different diseases.

**Teaching Process**

This study implements a tiered teaching process, divided into three stages: pre-class independent exploration, in-class group discussion, and post-class application and transfer.

1. **Pre-class Independent Exploration Stage**: Seven days before the class, teachers distribute CPC cases containing clinical data and tiered discussion questions to guide students in building their knowledge systems through literature searches and group collaborations. Key points that have not been covered in previous studies are highlighted for pre-learning. Teachers monitor discussion hotspots using online platforms, such as controversies over the differential diagnosis of pulmonary embolism and thrombosis, providing data support for classroom interventions.
2. **In-class Group Discussion Stage**: A 45-minute discussion session is set up, including:

3. **Group Representatives' Presentation** (8 minutes): Representatives present diagnostic evidence within a time limit.

①**Cross-examination and Debate** (e.g., arguing the source of the embolus): This encourages critical thinking and discussion among students.

②**Teacher-led Dynamic Guidance**: Teachers use "positive reinforcement strategies" to solidify correct understandings or employ the "evidence chain tracing method" to correct deviations. Three-dimensional animations are used to demonstrate pathological processes, establishing connections between morphology and function.

③**Post-class Consolidation and Application Stage**: A hierarchical homework system is designed. The basic level requires students to draw mind maps illustrating the progression of lesions, while the advanced level involves virtual clinical scenario decision-making tasks. A multi-faceted evaluation mechanism is established, incorporating the quality of classroom participation (30%), logical rigor (40%), and innovation (30%) into formative assessments. Feedback from assignments helps achieve a closed-loop teaching process.
