# Supplementary material for: Enhancing clinical reasoning skills through tailored CPC in pathology laboratory instruction
Source: Front Med (Lausanne). 2025 Jul 25;12:1566097. doi: 10.3389/fmed.2025.1566097 (PMC12331614; doi:10.3389/fmed.2025.1566097)
Supplement: Supplementary file 2 [file Data_Sheet_2.pdf]

Basic Level Clinical Case

1. Case Summary

A 35-year-old male was admitted to the hospital with a chief complaint of "recurrent anorexia, fatigue, jaundice, and right upper quadrant abdominal pain persisting for over one year." Five years prior, he was found to be HBsAg-positive during a routine physical examination; however, he did not undergo regular antiviral therapy. He reported a history of daily alcohol consumption, averaging approximately 100 grams per day. Over the past year, he experienced progressive symptoms, including loss of appetite, significant fatigue, worsening cutaneous and scleral icterus, dark-colored urine resembling soy sauce, and persistent dull pain in the right upper quadrant that intensified with physical activity. His condition showed no improvement following self-administration of unspecified hepatoprotective medications.

Physical Examination:

The patient exhibited signs of chronic illness and moderate jaundice involving the skin and sclera. Percussion tenderness was present in the hepatic region. The liver was palpable 2 cm below the right costal margin and 3 cm below the xiphoid process, with a firm yet smooth surface; no splenomegaly was detected.

Laboratory Tests:

Alanine aminotransferase (ALT): 260 U/L (Reference range: 8–40 U/L)

Aspartate aminotransferase (AST): 180 U/L (Reference range: 5–40 U/L)

Total bilirubin: 78 μmol/L (Reference range: 3.4–17.1 μmol/L)

Direct bilirubin: 42 μmol/L (Reference range: 0–6.8 μmol/L)

Indirect bilirubin: 36 μmol/L (Reference range: 1.7–13.5 μmol/L)

Albumin: 36 g/L (Reference range: 40–55 g/L)

Globulin: 34 g/L (Reference range: 20–30 g/L)

Albumin/globulin (A/G) ratio: 1.06 (Reference range: 1.5–2.5:1)

Serological Tests:

HBsAg (+), HBeAg (+), anti-HBc (+); Alpha-fetoprotein (AFP): 18 ng/mL (Reference value ≤ 20 ng/mL).

Abdominal Ultrasound:

Increased and coarse echogenicity of the liver parenchyma with uneven distribution; portal vein diameter within normal limits.

After receiving antiviral therapy for one year without significant symptom relief, ultrasound-guided liver biopsy was performed. Histopathological examination revealed widespread hepatocellular swelling with vacuolated cytoplasm, focal ballooning degeneration of hepatocytes, focal necrosis with lymphocytic infiltration, scattered apoptotic bodies, mild fibrous proliferation in the portal areas, and prominent lymphocytic infiltration. However, the hepatic lobular architecture remained intact.

Discussion Questions:

（1） Based on laboratory tests and histopathological results, determine the pathological type of hepatitis in this case.

（2） List the characteristic histopathological features of chronic viral hepatitis compared to acute ordinary hepatitis.

（3） Based on the pathological and laboratory tests, explain the pathophysiological mechanisms underlying the patient's jaundice and fatigue.

2. Knowledge Objectives

（1） Understand the pathological changes of chronic viral hepatitis, including: Hepatocellular degeneration (e.g., hydropic swelling)、Necrotic changes (e.g., spotty necrosis and piecemeal necrosis)、Inflammatory cell infiltration、Mild fibrous proliferation in the portal tracts.

（1） Recognize the clinical manifestations and serological characteristics of viral hepatitis, including: Common symptoms such as anorexia, fatigue, jaundice, and

right upper quadrant pain、Serological markers associated with hepatitis B virus infection.

3. Application Objectives

（1） Pathological Diagnosis and Differential Diagnosis:
Use histopathological features and clinical presentation to make a diagnosis and differentiate between chronic and acute forms of hepatitis. For example, the presence of piecemeal necrosis and portal fibrosis supports a diagnosis of chronic viral hepatitis rather than acute ordinary hepatitis.
（2） Integration of Pathology and Clinical Practice:
Apply knowledge of hepatic pathology to explain clinical manifestations. Elaborate on how inflammatory changes and hepatocyte damage lead to non-specific symptoms such as jaundice and fatigue.

Intermediate Level Clinical Case

1. Case Summary
A 57-year-old male presented with "right upper quadrant pain, abdominal distension, and weight loss for three months, exacerbated by hematemesis and melena for one week." He was diagnosed with chronic hepatitis B more than ten years ago but did not receive regular antiviral treatment or follow-ups; his mother also had hepatitis B.
In the past three months, he experienced persistent dull pain in the right upper abdomen, accompanied by a sharp decrease in appetite (food intake reduced by about two-thirds), aversion to oily foods, fatigue, decreased physical endurance, and weight loss from 70 kg to 55 kg. He also developed abdominal distension, bilateral lower limb pitting edema, jaundice of the skin and sclera, and darkening urine color. In the last week, he had frequent coughing, worsening abdominal distension (abdominal circumference

increased from 85 cm to 100 cm), urine output decreased to 300-400 ml/day, passed dark red bloody stools three times (each episode 100-200 g), and experienced dizziness and palpitations. Early morning coffee-ground vomiting occurred, approximately 500 ml.

Physical Examination:

Temperature: 37.8°C, Pulse: 110 beats/min, Respiratory rate: 22 breaths/min, Blood pressure: 90/60 mmHg.

Chronic illness appearance, severe jaundice, palmar erythema, spider angiomas. Scattered moist rales were heard in the lower right lung, heart rhythm was regular. Abdomen was distended with visible varicose veins, liver palpable 3 cm below the xiphoid process, hard with nodules, positive percussion tenderness, spleen palpable 5 cm below the costal margin, shifting dullness positive, bilateral lower limb severe pitting edema.

Laboratory Tests:

Peripheral blood showed reduced white cells, red cells, and platelets; significantly elevated bilirubin levels, AST and ALT, decreased albumin content; abnormal coagulation function, prolonged PT, reduced fibrinogen; serological tests HBsAg (+), anti-HBe (+), anti-HBc (+), HBV-DNA quantitative 5.6×10^6 IU/ml, AFP 1200 ng/ml (reference range $\leq$ 20 ng/mL). Renal function tests revealed elevated urea nitrogen and creatinine.

Upon admission, the patient was immediately placed on fasting and bed rest, and received cardiac monitoring and oxygen supplementation. Treatment included somatostatin to reduce portal pressure, omeprazole for gastric protection, hemostatic therapy, coagulation factor replacement, albumin infusion, and entecavir for antiviral management. Although initial hemostasis was achieved, the patient continued to experience fever and abdominal distension. On the 7th day of hospitalization, he experienced a recurrence of massive hemorrhage and died despite resuscitative efforts

due to hemorrhagic shock.

Autopsy Summary:

Severe emaciation, jaundice, abdominal distension, and lower limb edema.

The abdominal cavity contained 1000 ml of straw-colored fluid, with multiple 0.1-0.3 cm white nodules on the mesentery, greater omentum, and parietal peritoneum. The intestines and stomach contained large amounts of brown fluid.

The liver weighed 1800 g, measuring 25×16×8 cm, covered with 0.1-1 cm nodules, some showing central necrosis. Microscopically, normal lobular structures were replaced by numerous hepatocellular nodules with disorganized cell arrangement, absent or displaced central veins, surrounded by narrow connective tissue. Larger grayish-yellow nodules consisted of irregular cellular cords and nests, deeply stained nuclei, uniform morphology, richly vascularized (Figure1-2).

Figure 1

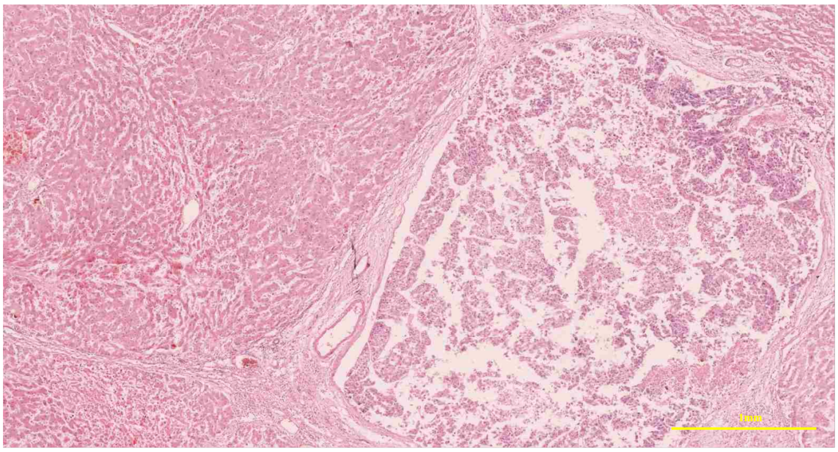

Figure 2

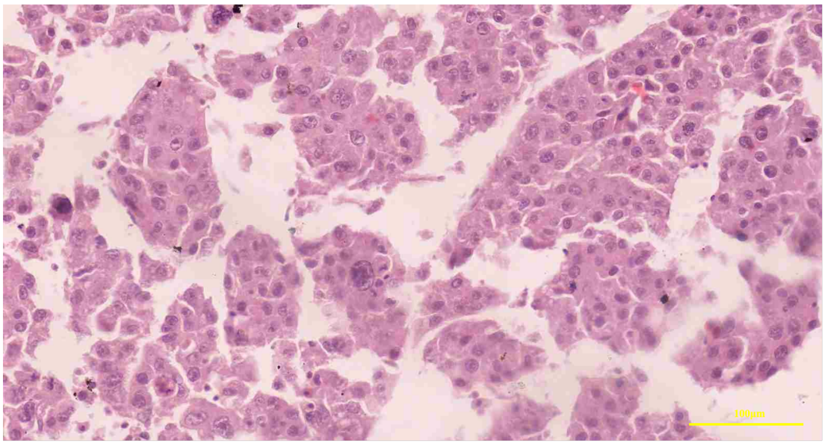

Lung surfaces and sections revealed multiple bean-to-walnut-sized gray-white round nodules. Microscopically, these areas were occupied by proliferative cells similar to those seen in the liver.

The spleen weighed 815 g, with tense capsule and dark red pulp. Microscopically, enlarged and congested splenic sinuses, splenic cords, and trabeculae proliferation were noted.

Esophagus showed dilated submucosal veins with small rupture sites.

Discussion Questions:

（1） Based on autopsy findings, identify the characteristic pathological changes of cirrhosis and hepatocellular carcinoma (HCC) and explain how to differentiate between them microscopically.

（2） Why are the pulmonary nodules considered metastatic HCC? Describe the hematogenous spread pathway and morphological evidence.

（3） List the clinical manifestations of portal hypertension in this case and analyze the causes of ascites formation.

（4） Briefly describe the evolution from chronic hepatitis B to cirrhosis to HCC and highlight key clinical and pathological evidence supporting this progression in this case.

（5） Propose at least three preventive measures against cirrhosis and HCC based on the patient's history and provide theoretical justifications.

2. Knowledge Objectives

（1） Understand the pathological changes and clinical manifestations of cirrhosis and HCC, such as recognizing pseudolobules in cirrhosis and diagnosing HCC through significant atypical tumor cells in biopsy specimens.

（2） Master the metastatic pathways of HCC (hematogenous spread to lungs, intrahepatic metastasis, implantation metastasis involving mesentery) and corresponding pathological features.

（3） Clarify the relationship and differences between cirrhosis and HCC in terms

of pathological morphology and clinical course development.

3. Application Objectives

（1） Accurate Pathological Diagnosis: Use the pathological characteristics of viral hepatitis, cirrhosis, and HCC to accurately diagnose cases; distinguish between primary and secondary lung tumors based on tumor morphology and metastatic patterns.

（2） Explain Clinical Mechanisms: Utilize hepatic pathology to elucidate mechanisms underlying clinical manifestations such as edema (due to portal hypertension and hypoalbuminemia) and upper gastrointestinal bleeding (from ruptured esophageal varices).

（3） Understand Pathogenesis and Prevention: Analyze the progression from chronic hepatitis to cirrhosis to HCC, comprehend pathogenic mechanisms, and propose preventive measures such as alcohol abstinence, antiviral therapy, and regular liver function monitoring based on cirrhosis etiology.

Advanced Level Clinical Case

1. Case Summary

A previously healthy 10-year-old female with no significant medical history of chronic illness, allergies, or recent illness, and with complete vaccination records, presented with a chief complaint of "persistent high fever for 20 days, a left-sided neck mass for one week, and generalized trunk petechiae for three days."

Approximately 20 days prior to admission, the child developed an acute high fever with temperatures consistently ranging between 39°C and 40°C, without associated symptoms such as chills, cough, or abdominal pain. The parents administered ibuprofen suspension for symptomatic relief, which resulted in temporary defervescence. However, the fever recurred shortly after each dose. Given the absence of other notable

symptoms, medical evaluation was initially delayed.

One week before admission, a firm, non-tender, 2–3 cm mass appeared on the left side of her neck. Over time, the lesion increased in size, became fluctuant, and developed tenderness, causing severe pain during swallowing and head movement. She was subsequently evaluated at a local hospital and diagnosed with cervical abscess. Incision and drainage were performed, yielding approximately 50 mL of thick yellow-green pus. Postoperatively, she received intravenous cefazolin sodium at a dose of 50 mg/kg/day, divided into three doses. Despite this intervention, the fever persisted, and the patient became increasingly lethargic and somnolent, with a documented weight loss of 2 kg.

In the last three days, multiple non-blanching petechiae appeared on the trunk. Due to unresolved fever and progressive clinical deterioration, she was referred to our hospital for further evaluation and management.

Physical Examination:

Temperature: 39°C, Pulse: 140 beats/min, Respiratory Rate: 36 breaths/min, Blood Pressure: 80/50 mmHg.

Cardiac auscultation revealed rapid and weak heart rate, grade III systolic murmur over the apex. Liver palpable 3 cm below the right costal margin along the midclavicular line and 4 cm below the xiphoid process, moderate consistency.

Laboratory Tests:

Complete Blood Count (CBC):

White blood cell count: 12×10^9/L (reference range: $4 - 10 \times 10^9$/L)

Red blood cell count: 3.07×10^{12}/L (reference range for female children: $3.5 - 5.0 \times 10^{12}$/L)

Hemoglobin: 85 g/L (reference range for female children: 110 – 160 g/L)

Platelet count: 80×10^9/L (reference range: $100 - 300 \times 10^9$/L)

Neutrophils: 89% (reference range: 50% – 70%)

Lymphocytes: 9% (reference range: 20% – 40%)

Basophils: 2% (reference range: 0% – 1%)

Biochemistry:

C-reactive protein (CRP): 180 mg/L (reference range: 0 – 8 mg/L)

Coagulation Profile:

Prothrombin time (PT): 16 seconds (reference range: 11 – 13 seconds)

Fibrinogen (FIB): 1.5 g/L (reference range: 2.0 – 4.0 g/L)

Blood Culture:

Peripheral venous blood was collected immediately upon admission. Three days later, culture results showed growth of Gram-positive cocci, consistent with Staphylococcus species.

Course of Treatment:

Upon admission, the child's condition deteriorated rapidly, showing signs of severe sepsis. Despite aggressive antibiotic therapy and fluid resuscitation, there was no improvement. On the fifth day of hospitalization, respiratory and cardiac arrest occurred, resulting in death.

Autopsy Summary:

Left neck showed a 2 cm incision, probing revealed a sinus tract about 3 cm long extending upwards into subcutaneous tissue and muscle layers towards the parapharyngeal space. Microscopically, the sinus walls consisted of necrotic tissue and numerous degenerated neutrophils, deeper layers contained granulation tissue with extensive neutrophil infiltration.

Heart: Large amounts of purulent exudate within the pericardial cavity, thickened and rough epicardium. Opening the heart chambers revealed two yellow lesions on the left

ventricular wall surrounded by dark red inflammatory zones. Cutting open these lesions released pus. A 0.5 cm grayish-white vegetation was observed on the posterior mitral valve leaflet, which had formed a perforation. Microscopically, myocardial necrosis and abundant neutrophil infiltration were noted, along with Gram-positive cocci.

Liver: Swollen and dull appearance, multiple scattered yellow lesions visible. Microscopic examination revealed dilated central veins and hepatic sinusoids filled with red blood cells, areas of hepatocyte necrosis, and significant neutrophil infiltration (figure 3-4).

Figure 3

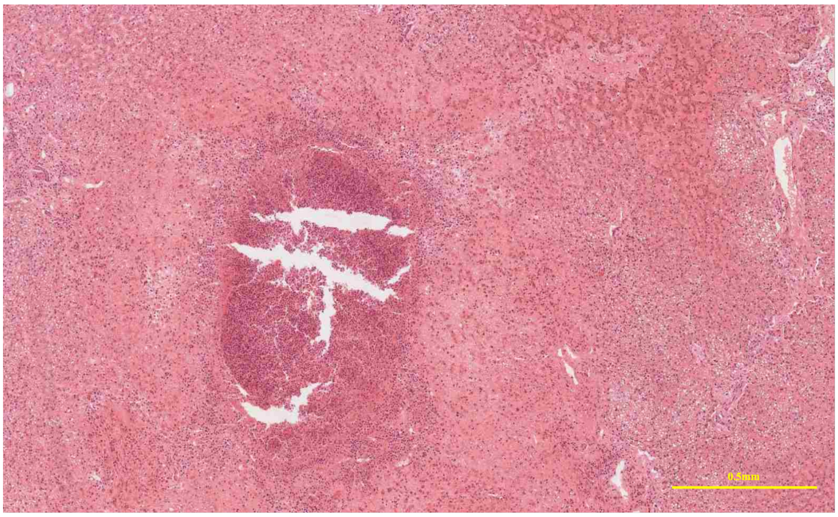

Figure 4

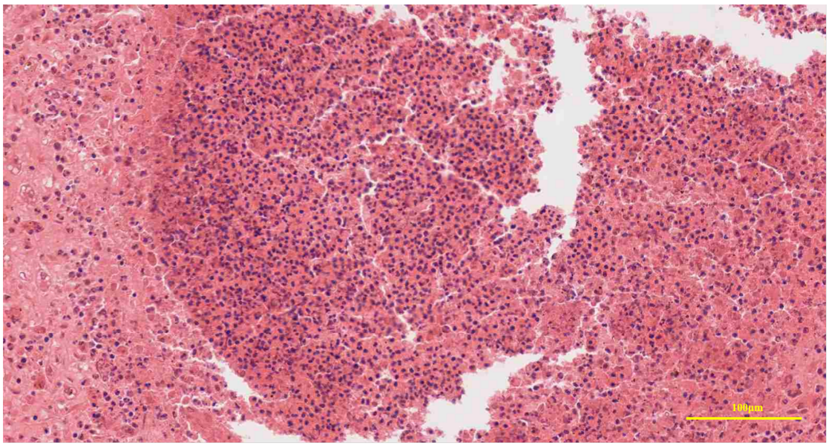

Lungs: Yellowish exudates on the pleura of the right lung, both lungs enlarged and reddened. A 2×2 cm irregular cystic cavity containing thick yellow fluid was found in the lower lobe of the right lung. Microscopically, alveolar septa were congested and

edematous, with large numbers of degenerated neutrophils and bacterial colonies present.

Kidneys: A 0.3 cm solid material was seen in the left renal artery, not adherent to the vessel wall. A wedge-shaped lesion measuring about 3×2 cm was observed under the capsule, with dark red margins around it (figure 5). Microscopically, necrosis of renal tissue and visible glomerular and tubular structures were noted (figure 6).

Figure 5

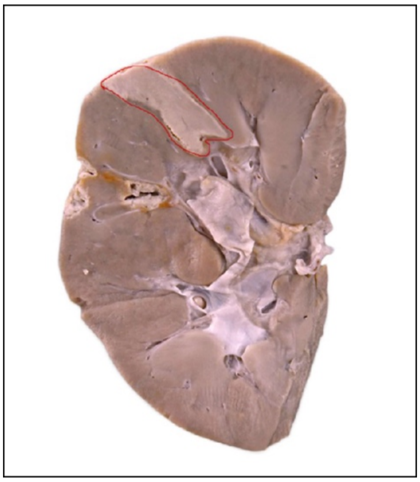

Figure 6

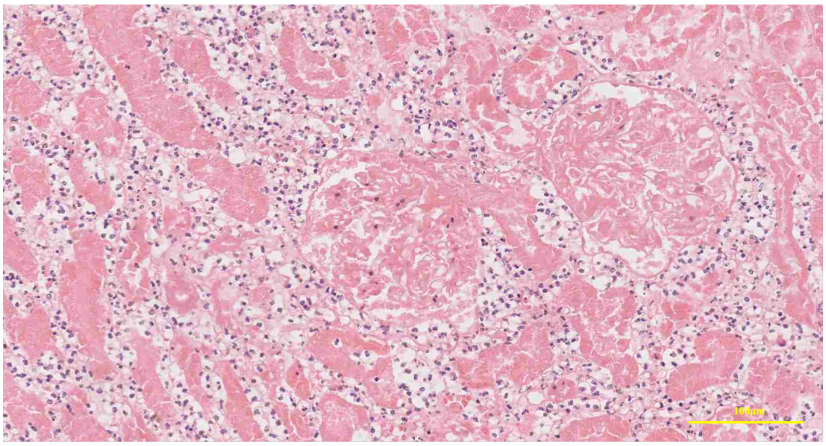

Brain and Meninges: A 3.5 cm long dark red solid material was found in the sigmoid sinus. Microscopy showed hemorrhage in the ventricles and subarachnoid space (figure 7).

Figure 7

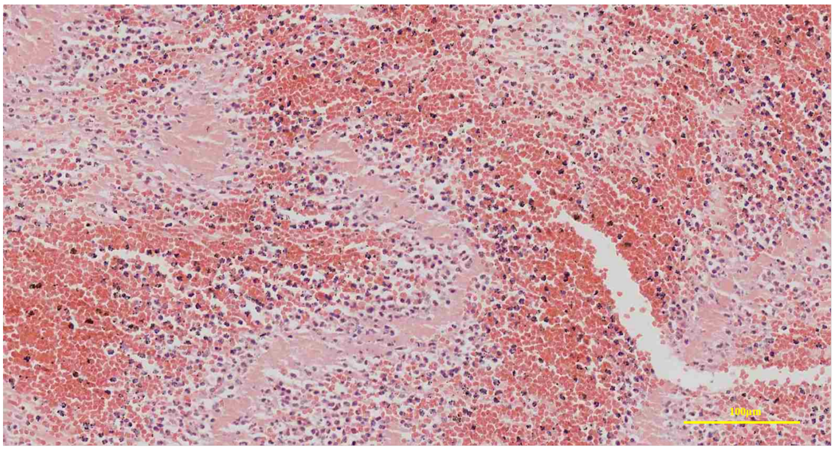

Discussion Questions:

（1） Based on clinical presentation, Laboratory Tests, and autopsy results, what are the complete pathological diagnoses for this patient?

（2） What type of inflammation does the mitral valve vegetation and perforation represent? How do these changes affect cardiac function?

（3） Compare the pathological features of acute and subacute infective endocarditis. Explain why this case fits the diagnosis of acute infective endocarditis based on pathological evidence.

（4） Analyze the causes of cardiac murmurs and liver enlargement.

（5） Where did the embolus in the renal artery originate from? What category does this infarction belong to? Discuss the pathologic connection between thrombosis, embolism, and infarction.

（6） Considering conditions promoting thrombus formation, analyze the reasons for sigmoid sinus thrombosis.

（7） Briefly describe the entire course of disease development in this case.

（8） Given that “aggressive antibiotics and fluid resuscitation” failed to save the child, analyze potential reasons for ineffective treatment based on the characteristics of sepsis, septic shock, and infective endocarditis, and suggest more effective treatment strategies.

2. Knowledge Objectives

（1） Understand the classification and lesion characteristics of suppurative

inflammation.

（2） Master the lesion characteristics of sepsis and septic shock, distinguish between pathological changes and clinical manifestations during different stages of the disease progression.

（3） Grasp the pathological changes of acute infective endocarditis and clarify its differences from subacute infective endocarditis.

（4） Comprehend the pathology of thrombosis, embolism, and infarction, understand the mechanisms involved in kidney and brain vascular thrombosis leading to infarction in this case.

3. Application Objectives

（1） Comprehensive Diagnosis and Differential Diagnosis: Accurately diagnose based on autopsy results combined with clinical presentations and lab tests.

（2） Pathological Mechanism Analysis and Reasoning: Analyze how bacterial infection leads to a series of pathological processes including suppurative inflammation, sepsis, septic shock, thrombosis, embolism, and infarction.

（3） Evaluation and Reflection on Treatment Plans: Evaluate why aggressive antibiotics and fluid resuscitation were ineffective, consider more appropriate treatment strategies.

（4） Clinical Risk Prediction and Intervention: Use knowledge about thrombosis, embolism, and infarction to predict and intervene early in potential complications.
